# A MultiSite Gateway Toolkit for Rapid Cloning of Vertebrate Expression Constructs with Diverse Research Applications

**DOI:** 10.1371/journal.pone.0159277

**Published:** 2016-08-08

**Authors:** Daniel K. Fowler, Scott Stewart, Steve Seredick, Judith S. Eisen, Kryn Stankunas, Philip Washbourne

**Affiliations:** 1 Institute of Molecular Biology, Department of Biology, University of Oregon, Eugene, Oregon, United States of America; 2 Institute of Neuroscience, Department of Biology, University of Oregon, Eugene, Oregon, United States of America; Imperial College London, UNITED KINGDOM

## Abstract

Recombination-based cloning is a quick and efficient way to generate expression vectors. Recent advancements have provided powerful recombinant DNA methods for molecular manipulations. Here, we describe a novel collection of three-fragment MultiSite Gateway cloning system-compatible vectors providing expanded molecular tools for vertebrate research. The components of this toolkit encompass a broad range of uses such as fluorescent imaging, dual gene expression, RNA interference, tandem affinity purification, chemically-inducible dimerization and lentiviral production. We demonstrate examples highlighting the utility of this toolkit for producing multi-component vertebrate expression vectors with diverse primary research applications. The vectors presented here are compatible with other Gateway toolkits and collections, facilitating the rapid generation of a broad range of innovative DNA constructs for biological research.

## Introduction

Most contemporary investigations of cellular and molecular processes necessitate the use of synthetic DNA vectors. Recombinant cloning of plasmid vectors is the most commonly used method for transgenic analyses. Shortly after the first successful demonstration of gene expression from exogenous DNA in mammalian cells [1], synthetic vectors were established as a powerful method to assay gene function *in vitro* and *in vivo*. Over time, the development of sophisticated techniques such as genetic knockdown and knockout allowed more intricate and detailed investigations. Today, the continued advancement of recombinant DNA technologies has provided the modern biologist with an arsenal of molecular tools. Use of these techniques, however, often requires the laborious construction and validation of complex, multi-component vectors.

The effort associated with conventional cloning methods still prevents many researchers from exploiting recombinant DNA tools. Plasmids are often still constructed with traditional “cut-and-paste” restriction enzyme techniques that are difficult and time consuming. Moreover, restriction enzyme-based cloning methods are prohibitive for DNA sequences containing common endogenous restriction sites. This problem is exacerbated when insertion of more than one DNA sequence into a vector is desired. The MultiSite Gateway^TM^ cloning system (ThermoFisher) utilizes site-specific recombination to insert DNA elements into a vector [2] and has been established as a fast and efficient alternative for generating multi-component plasmids [3]. Further, molecular “toolkits” have been created for the Gateway system that provide modular DNA elements for specific applications, such as fluorophores for imaging and methods for genetic manipulation [4–18]. Additionally, genome-wide open reading frame libraries (ORFeomes) containing protein coding sequences from human [19–22], worm [23, 24], frog [25] and multiple bacteria [26–29] have been cloned into Gateway-compatible vectors, representing valuable resources for the characterization of individual genes.

Here, we present a novel set of three-fragment MultiSite Gateway vectors which provide an expanded array of molecular tools. Our entry vectors cover a large variety of applications such as cell-specific expression, fluorophore-based imaging, bicistronic expression, inhibitory RNA (RNAi)-mediated gene knockdown, protein purification and inducible protein dimerization. Additionally, we created two destination vectors for lentiviral production and describe optimized entry vectors designed for enhanced viral titers.

We illustrate the diverse uses of these vectors with examples from multiple primary research applications. Using an entry vector with a novel brainbow color palette, we label motoneuron circuitry in zebrafish. Next we demonstrate efficient artificial microRNA (amiRNA)-mediated knockdown of multiple genes in primary neuronal cultures using lentivirus produced from vectors containing our recently enhanced amiRNA expression scaffold [30]. We then show high-fidelity bicistronic protein expression using vectors containing the porcine teschovirus-1 2A (P2A) “self-cleaving” peptide [31]. Finally, we demonstrate the use of vectors for tandem affinity protein purification and for rapamycin-induced protein dimerization. All of our vectors are available through Addgene (www.addgene.org). Together, the tools presented here will prove useful for cutting-edge molecular and genetic investigations.

## Materials and Methods

### Vertebrate animals

Studies using rats or zebrafish were carried out in strict accordance with the recommendations in the Guide for the Care and Use of Laboratory Animals of the National Institutes of Health. The protocols were approved by the University of Oregon Institutional Animal Care and Use Committee (Permit Numbers: #13–19 and #11–20). Rats were anesthetized with isoflurane prior to cervical dislocation and culturing of neurons. Zebrafish embryos were anesthetized with MS-222 prior to embedding in agarose and imaging. All zebrafish embryos, larvae and adults were raised and maintained at 28.5°C with a 14/10 light/dark cycle according to standard protocols [32] in the University of Oregon Zebrafish Facility. All zebrafish were of the AB line. Rats were housed with a 12/12 light/dark cycle according to standard protocols in the University of Oregon Animal Care Facility. Sprague Dawley rats were obtained from Envigo (Indianapolis, IN).

### Vector design and cloning

#### 5’ entry vectors

Unless otherwise stated, all 5’ entry vectors were generated by PCR amplification of the desired 5’ element using attB4/B1R-flanked oligonucleotide primers, followed by a BP reaction with pDONR P4-P1R (Invitrogen). p5E-CMVmin was created by amplification of the minimal cytomegalovirus immediate early enhancer/promoter (CMVmin) from pcDNA3 (Invitrogen). The human synapsin1 promoter alone or hybrid CMVmin/human synapsin1 promoter from pENTR-L1-ESYN-R5 [12] were amplified to make p5E-hSyn1 and p5E-ESyn1, respectively. To make p5E-hPGK, an EcoRV fragment containing the human PGK promoter from pLenti PGK PURO DEST [4] was cloned into the Klenow-blunted AscI site of p5EM-FA, a modified version of p5E-Fse-Asc [6] with extraneous sequences removed. For p5E-Ui4-eSIBR, a PmeI/BamHI Klenow-repaired Ui4-SIBR cassette from pUi4-GFP-SIBR [33] was inserted into the Klenow-blunted AscI site of p5EM-FA; the wild-type SIBR cassette was then swapped for the eSIBR cassette [30]. p5E-*elavl3* was made by inserting a XhoI-containing linker into the NotI site of a *huC*:*Cam2*.*1* plasmid [34] and cloning the 8723-nucleotide XhoI fragment into the XhoI site of p5E-MCS [6]. p5E-*gfap* was made by cloning a 7437-nucleotide XhoI-BamHI fragment from a *gfap*:GFP plasmid [35] into p5E-MCS.

The generation of p5E-EF1α/β-globin and p5E-*krt5* [36], p5E-*mnx1* [37] and p5E-*dusp6* [38] have been previously described. p5E-EF1α/β-actin was made by cloning the 1714-nucleotide SalI fragment of p5E-*bactin2* [6] into the SalI site of p5E-EF1α/β-globin.

#### Middle entry vectors

Unless otherwise stated, all middle entry vectors were generated by PCR amplification of the desired middle element using attB1/B2-flanked oligonucleotide primers, followed by a BP reaction with pDONR221 (Invitrogen). To create pME-mKate2 no-stop, the mKate2 coding sequence [39] was amplified from pmKate2-C (Evrogen) with the 5’ primer additionally containing a Kozak sequence. pME-tdTomato was generated by cloning a 1507-nucleotide BglII-XbaI fragment containing an optimized Kozak sequence, the tdTomato ORF [40] and 3’ elements into a BamHI-XbaI fragment of pME-MCS [6]; the no stop version with Kozak sequence was then amplified and inserted into pDONR221. pME-BrainbowTEC was generated sequentially. First, a Brainbow1.0 recombination scaffold including nested *loxP* and *lox2272* sites and 3 SV40 polyadenylation sequences was created by PCR. This 1024-nucleotide recombination scaffold was cloned into KpnI-SacI sites of pME-MCS. Then HA-tagged E2Crimson (Clontech), Myc-tagged tdTomato and EGFP were cloned in sequence into unique PacI, AscI and FseI sites within the recombination scaffold, respectively. pME-FlEx was created by annealing sets of oligonucleotides to produce antiparallel tandem *loxP* and *lox2272* recombination sites which was then PCR amplified inserted into pDONR221.

To generate P2A middle entry vectors, the GFP, nlsGFP, and memGFP sequences were first subcloned into pcDNA3. Sequences for GFP or nlsGFP with Kozak sequences and without stop codons were amplified from pME-nlsEGFP [6] and inserted between the HindIII and BamHI sites to make pcDNA3-GFP no stop and pcDNA3-nlsGFP no stop. To make pcDNA3-memGFP no stop, the memGFP sequence without a stop codon was generated by amplification of GFP using a 5’ primer containing a Kozak sequence and the Fyn myristoylation sequence [41], followed by insertion between HindIII and BamHI sites of pCDNA3. Next, annealed sense and antisense oligonucleotides containing the P2A sequence [42] and 5’ overhangs were inserted between BamHI and NotI to make pcDNA3-GFP-P2A and pcDNA3-nlsGFP-P2A, or between EcoRI and NotI to make pcDNA3-memGFP-P2A. Both restriction sites used for insertion of the P2A sequence were destroyed upon ligation for clonal screening purposes. Finally, sequences including the Kozak consensus were amplified from pcDNA3-GFP-P2A, pcDNA3-nlsGFP-P2A and pcDNA3-memGFP-P2A and recombined by BP reaction to generate pME-GFP-P2A, pME-nlsGFP-P2A and pME-memGFP-P2A, respectively.

The generation of pME-eSIBR [30], pME-ERT2-Cre-ERT2 [38] and pME-Gal4-ERT2-VP16 [36] have been previously described

#### 3’ entry vectors

Unless otherwise stated, all 3’ entry vectors were generated by PCR amplification of the desired 3’ element using attB2R/B3-flanked oligonucleotide primers, followed by a BP reaction with pDONR P2R-P3 (Invitrogen). p3E-GFP-HA, p3E-YFP-HA (from pEYFP-C1, Clontech), p3E-CFP-HA (from pECFP-C1, Clontech), p3E-mCherry-HA (from p3E-mCherrypA [6]), p3E-mKate2-HA and p3E-mKate2-myc no-polyadenylation signal (pA) were made by amplification of the coding sequences without a stop codon, but with the 3’ primer additionally containing an HA or c-myc epitope sequence followed by a stop codon. p3E-HA-Neuroligin1 was generated by amplification of HA-Neuroligin1 from a previously described vector [30]. p3E-Dam-myc no-pA is from pNDam-Myc [43]. p3E-SGTAP no-pA originated from pCeMM-CTAP(SG)-Gw [44]. p3E-FRT-Kan^R^-FRT pA was cloned by amplifying the Kan^R^ cassette from pCRII (ThermoFisher) with FRT sites in the 5’ and 3’ primers and inserting into the BamHI site of p3E-polyA [6]. FRB variants and FKBP cassettes were amplified from Gerald Crabtree lab plasmids [45–47] to generate p3E-FRB-HA, p3E-FRB(PLF)-HA, p3E-FRB(KTF)-HA, p3E-FKBP-HA and p3E-mCherryFRB-HA no-pA. p3E-HA was made by cloning annealed oligos (5’-GATCCACTCGAGTATCCGTACGACGTACCAGACTACGCAGCATAGGGTACCAGTACTAAC-3’ and 5’-AATTGTTAGTACTGGTACCCTATGCTGCGTAGTCTGGTACGTCGTACGGATACTCGAGTG-3’) into BamHI/MfeI-digested p3E-polyA. This element provides an HA tag, a stop codon, additional cloning sites and lacks a pA sequence.

GFP, nlsGFP, GFPmem and CMVmin-promoted 3’ entry vectors were generated by first subcloning GFP, nlsGFP, or GFPmem coding sequences into pcDNA3. GFP and nlsGFP were amplified from pME-nlsGFP and inserted between HindIII/NotI to make pcDNA3-GFP and pcDNA3-nlsGFP. GFPmem, which contains the palmitoylation domain of human H-Ras on the c-terminus, was amplified from pME-EGFPCAAX [6] and inserted between HindIII/EcoRI to make pcDNA3-GFPmem. Sequences from these intermediates were then amplified to generate entry vectors: 1) for no-pA constructs, the 3’ primer annealed to the native stop codons, 2) for pA constructs the 3’ primer encompassed the bovine growth hormone polyadenylation sequence (BGHpA) of pcDNA3, and 3) for CMVmin-promoted constructs, a 5’ primer was used that annealed 242 nucleotides upstream of the CMVmin promoter of pcDNA3 to provide an “insulating” space between an independently-promoted open reading frame positioned upstream of the CMVmin promoter.

To make P2A 3’ entry vectors, the MCS from pBluescript II SK(+) (Agilent) was amplified to make p3E-MCS. Sense and antisense oligonucleotides containing the P2A sequence were annealed and inserted into the KpnI site of p3E-MCS to create p3E-P2A-MCS, which has unique restriction enzyme sites downstream of the P2A sequence in the 5’ to 3’ order of: XhoI, SalI, AccI, ClaI, HindIII, XmaI, SmaI, SpeI, BstXI and SacII. Coding sequences for GFP, CFP, and mKate2 were then amplified and inserted between XhoI/HindIII of p3E-P2A-MCS to produce p3E-P2A-GFP, p3E-P2A-CFP and p3E-P2A-mKate2 no-pA.

#### Destination vectors

pEpic was made by blunt-end cloning the XhoI/ClaI-defined attR4-attR3 cassette from pDestTol2pA2 [6] into the 7.3kb EcoRV fragment of pLenti PGK PURO DEST, effectively replacing its attR1-attR2 cassette. pEpic_Lite was created by removing the Puro^R^ cassette by AgeI/ApaI restriction enzyme digestion, filling in 5’ overhangs with DNA polymerase I Klenow fragment, and blunt-end ligation with T4 DNA ligase.

#### Additional vector sources

Tol2kit vectors pDestTol2CG2, pDestTol2pA2, p5E-UAS, p5E-CMV/SP6, p5E-Fse-Asc, p5E-*bactin2*, p5E-MCS, p3E-polyA, p3E-mCherrypA, p3E-MTpA, pME-EGFPCAAX, pME-MCS and pME-nlsEGFP [6] were generous gifts from Chi-Bin Chien and Kristen Kwan. pLenti PGK PURO DEST was made by Eric Campeau’s lab and was acquired through Addgene (plasmid #19068). pUi4-GFP-SIBR was a gift from David Turner. pNDam-Myc originates from Steven Henikoff’s lab. pCeMM-CTAP(SG)-Gw was acquired through the EuroSCARF plasmid repository (#30534). The *huC*:*Cam2*.*1* plasmid was a gift from Joe Fetcho. The *gfap*:GFP plasmid was generously provided by Pam Raymond. pDONR223-ErbB3 and pDONR223-ErbB2 [48] were gifts from William Hahn and David Root (Addgene plasmids #23874 and #23888). pENTR-L1-ESYN-R5 was provided by Matthew Nolan (Addgene plasmid #32581). pME-OGT1 and pME-Baf57c contain full length human OGT1 and human Smarce1 (Baf57c) open reading frames, respectively, without stop codons cloned into pDONR221. They were acquired from the DNASU plasmid repository (clones HsCD00042534 and HsCD00039580).

#### LR recombination reactions

Vectors were generated using recombination reactions with LR-clonase II (Invitrogen). UAS:BrainbowTEC was made by combining p5E-UAS, pME-BrainbowTEC, p3E-polyA, and pDestTol2CG2. pEpic_Lite mCMV:eSIBR-nlsGFP pA or no-pA vectors were created by combining pEpic_Lite, p5E-CMVmin, pME-eSIBR constructs with pre-inserted amiRNAs, and p3E-nlsGFP pA or no pA, respectively. pEpic_Lite UbiC:eSIBR-nlsGFP no-pA vectors were produced by combining pEpic_Lite, p5E-Ui4-eSIBR constructs with pre-inserted amiRNAs, pME-nlsGFP, and p3E-HA no-pA. pEpic_Lite mCMV:ErbB3-P2A-GFP was made by combining pEpic_Lite, p5E-CMVmin, pDONR223-ErbB3, and p3E-P2A-GFP no-pA. ErbB2-myc was created by combining pEpic_Lite, p5E-CMVmin, pDONR223-ErbB2, and p3E-MTpA. pEpic_Lite mCMV:memGFP-P2A-HA-Neuroligin1 was made by combining pEpic_Lite, p5E-CMVmin, pME-memGFP-P2A, and p3E-HA-Neuroligin1. pEpic hPGK:Baf57c-SGTAP was constructed by combining pEpic, p5E-hPGK, pME-Baf57c, and p3E-SGTAP no-pA. pEpic CMV:OGT1-mCherry-FRB-HA was made by combining pEpic, p5E-CMV/SP6 [6], pME-OGT1, and p3E-mCherryFRB-HA no-pA.

### Generation of UAS:BrainbowTEC zebrafish

Transgenic UAS:BrainbowTEC lines were generated by co-injecting plasmid DNA and Tol2 transposase RNA [49] into the yolk of one-cell stage embryos. Multiple founders were recovered and characterized. The founders used for this study were selected for multiple insertions giving rise to mixed fluorescent protein expression, and strong expression, with a low degree of mosaicism. Embryos carrying transgenic insertions (*cmlc*:GFP*-*positive) produced from these founders were indistinguishable from siblings lacking transgenic insertions (*cmlc*:GFP*-*negative).

### UAS:BrainbowTEC labeling of primary motoneurons

To label neurons in the ventral spinal cord of zebrafish embryos, Tg(*mnx1*:GAL4)^b1222^; Tg(*hsp70l*:cre)^zdf13^ were crossed to Tg(UAS:BrainbowTEC) founders. To induce Cre expression, embryos at 6–7 hpf were heat-shocked for 30 min by transfer into embryo medium pre-heated to 38.5°C as optimized previously [50]. 48–52 hpf embryos were agar mounted and the mid-trunk region of the spinal cord adjacent to somites 8–15 live imaged using a 40x water immersion objective on a Zeiss Pascal confocal microscope. The brightness and contrast of images was adjusted using Photoshop CS5 (Adobe).

### Cell culture, lentiviral production and titration

COS7 and HEK293T cell (ATCC^®^ cat # CRL-1651 and CRL-3216, respectively) culture and transfection, and production and titration of lentivirus was previously described [30]. For nlsGFP expression comparisons, 20,000 HEK293T cells were plated per well of a 12-well plate and transduced with lentivirus at single-particle levels (5–20% transduction), and cells were used for flow cytometry 5 days later. For ErbB3 phosphorylation experiments, 1 μg each of pEpic_Lite mCMV:ErbB3-P2A-GFP and pEpic_Lite mCMV:ErbB2-myc were transfected into COS7 cells per well of a 12-well plate. 48 hours later cells were harvested for western blotting or treated with 10 nM recombinant human neuregulin1-β (Reprokine) for 5 minutes and then harvested. For HA-Neuroligin1 experiments, COS7 cells were transfected with 1 μg of pEpic_Lite mCMV:memGFP-P2A-HA-Neuroligin1 per well of a 12-well plate and used 48 hours later for western blotting or immunocytochemistry. For SGTAP experiments, HEK293T cells were transduced with lentivirus carrying pEpic CMV-Baf57c-SGTAP. Transduced cells were selected with 1μg/ml puromycin.

### Primary hippocampal neurons

Primary rat hippocampal cell cultures were prepared and maintained as previously described [30]. For saturating transduction with lentivirus, 20,000 infectious lentiviral particles (as calculated by our titration method) were added per well of a 12-well plate at 2 days *in vitro* (DIV); for sub-saturating transduction 2,000 infectious lentiviral particles were added. Cells were used for western blotting, qRT-PCR, or immunocytochemistry at 14DIV.

### Flow cytometry

Single-cell GFP intensity was measured by flow cytometry on an Attune^®^ acoustic focusing cytometer (Applied Biosystems). GFP+ cells were determined as cells with >2x the maximum signal observed from non-transduced sister cultures for the BL1 channel (488 nm excitation, 530/30 nm emission filter). Mean BL1 values of all GFP+ cells in a culture were used for comparisons.

### Western blotting

Standard SDS-PAGE western blotting procedures using nitrocellulose membranes were performed as previously described [30]. Chemiluminescent blotting was performed with primary antibodies for ErbB3 phospho-Y1289 (rabbit clone 21D3, 1:1000, Cell Signaling), GFP (chicken, 1:2000, Aves Labs) and streptavidin binding protein (mouse, 1:1000, Santa Cruz); secondary antibodies used were anti-rabbit or anti-chicken HRP (donkey, 1:5000, Jackson ImmunoReserarch) or anti-mouse HRP (donkey, 1:10000, Cell Signaling); blots were developed using ECL Plus reagents (Pierce). Two-color, near-infrared blotting was performed using primary antibodies for HA.11 (mouse clone 16B12, 1:2000, BioLegend) and GFP (chicken, 1:2000, Aves Labs) and using secondary anti-mouse IRDye 680RD and anti-chicken IRDye800CW antibodies (donkey, 1:1000, LI-COR); blots were imaged on an Odyssey-Fc imaging system (LI-COR). Quantitative western blotting using Cadm1, Cadm3 and Cadm1-3 antibodies has been previously described [30]. The contrast and intensity of representative blot images were adjusted in GIMP (The GIMP Team, www.gimp.org).

### qRT-PCR

First-strand cDNAs were synthesized from total RNA isolated from cultured hippocampal neurons using oligo-dT primers and qRT-PCR was performed as described previously [30]. Values and relative expression levels were compared using the ΔΔC_t_ method. Primer sets used to measure mRNA levels were: nlgn1 (forward: GCACACTGACTTGGATCACG, reverse: TGGGAATCATTGTGATGGTG), nlgn2 (forward: CGTAAGACCCTGTTGGCACT, reverse: ACACCAAAGACGTAGGGCAG), nlgn3 (forward: GGAAGTAGCCTGGTCCAAATACA, reverse: GATCACGAACCCTTGGTTTCA).

### Immunocytochemistry and imaging

For COS7 cells and primary hippocampal neurons, cells grown on circular glass coverslips (ThermoFisher) were fixed in 4% PFA and 4% sucrose in 1X PBS for 15 min at 4°C, rinsed 1X with PBS, and blocked for 1 hr at RT in 1X blocking buffer (10% BSA (Sigma Aldrich), 1X blocking reagent (Roche) and 1% normal donkey and goat serums (Jackson ImmunoResearch) in 1X PBS). Cells were then incubated with a primary antibody for HA.11 (mouse clone 16B12, 1:1000, BioLegend) in 0.33X blocking buffer diluted in 1X PBS for 2 hr at RT. Cells were then washed 3 x 5 min with 1X PBS, permeabilized with 0.25% Triton X-100 in 1X PBS for 5 min at RT, then re-blocked with 1X blocking solution for 1 hr at RT. Cells were then incubated with primary antibodies overnight at 4°C; GFP (chicken, 1:2000, Aves Labs) and neurons additionally used Synapsin1 (rabbit, 1:500, EMD Millipore). The following day cells were rinsed 3 x 5 min with 1X PBS and incubated for 1 hr at RT with secondary antibodies; anti-chicken Alexa Fluor 488 (goat, 1:500, Jackson ImmunoResearch) and anti-mouse Cy3 and anti-rabbit Cy5 (donkey, 1:500, Jackson ImmunoResearch). Cells were washed 3 x 5 min in 1X PBS and mounted on slides with Fluoromount-G (Southern Biotech). Images were taken using a 40X air or 100X oil-immersion objective on an inverted Nikon Eclipse C1 confocal microscope. The brightness and contrast of images was adjusted using GIMP. The binary GFP mask was produced by intensity thresholding of the GFP image in Image-Pro Plus 6.3 (Media Cybernetics).

### SGTAP purification

Nuclear extracts [51] were prepared from lentivirally-transduced HEK293T cells and used to purify Baf57c using SGTAP as described previously [44], except that Baf57c was directly eluted from sepharose beads with SDS sample buffer without a biotin-elution step. Samples at various stages of the purification were immuno-blotted using standard SDS-PAGE methods.

### Rapamycin-induced dimerization

HEK293T cells were co-transfected with 500 ng each of pEpic CMV:OGT1-mCherry-FRB-HA and pS-FKBPNES (which expresses human FKBP12 fused to the nuclear export sequence from HIV REV protein) per well of a 24-well plate using Xfect transfection reagent (Clontech). 24 hours later, the cells were treated with 50 nM rapamycin (LC Labs) and time-lapse imaged using a Nikon Eclipse Ti–E fluorescent inverted widefield microscope equipped with a LiveCell environmental control system (Pathology Devices).

### Statistical analysis

P-values obtained by statistical comparisons of two sample groups with normal distributions verified by Shapiro-Wilk tests for normality in R (R Foundation for Statistical Computing, Vienna, Austria) used Student’s two-tailed, unpaired t-tests in Microsoft Excel. Sample group variances were compared using F-tests in R, and comparisons with equal variances used type 2 (homoscedastic) assumptions, whereas comparisons with unequal variances used type 3 (heteroscedastic) assumptions.

## Results and Discussion

### Introduction to MultiSite Gateway cloning and overview of toolkit vectors

**Fig 1:**
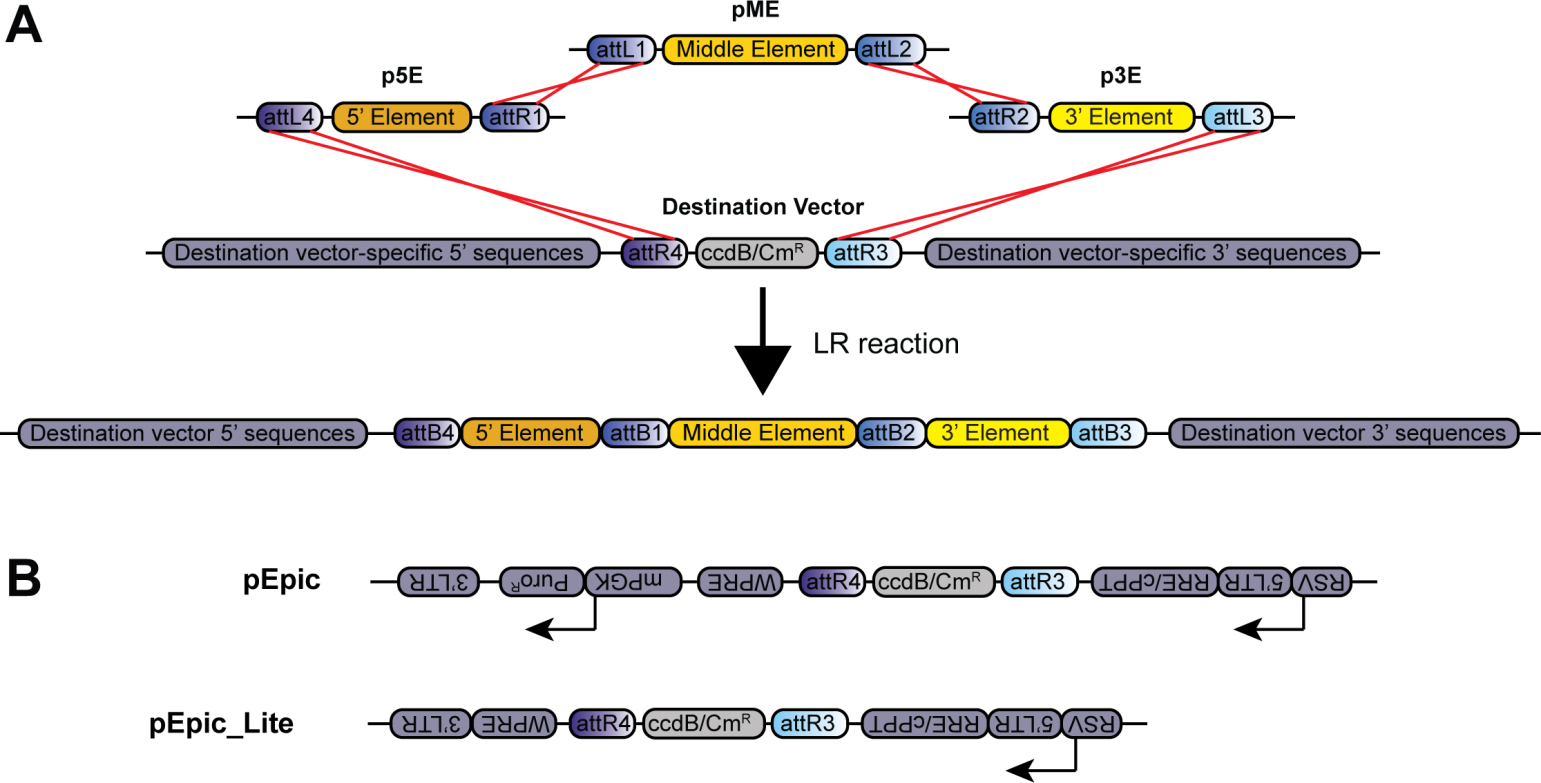
Overview of three-fragment MultiSite Gateway cloning and novel lentiviral destination vectors. (A) Schematic of an LR recombination reaction and the resulting vector. Site-specific recombination events (red lines) between attR and attL sites from a 5’, middle, and 3’ entry vector with a destination vector replaces the ccdB/Cm^R^ selection cassette of the destination vector with the mobile DNA elements from the entry vectors, leaving destination vector-specific 5’ and 3’ sequences intact. (B) Schematic of lentiviral destination vectors pEpic and pEpic_Lite. attR3 and 4 sites flanking the ccdB/Cm^R^ selection cassette are positioned in an anti-sense orientation to viral RNA expression driven by a Rous sarcoma virus (RSV) promoter. pEpic_Lite lacks puromycin resistance (Puro^R^). LTR = long terminal repeat; RRE = Rev response element; cPPT = central polypurine tract; ccdB = E. coli ccdB toxin; Cm^R^ = chloramphenicol resistance; mPGK = mouse phosphoglycerate kinase promoter; WPRE = woodchuck hepatitis virus posttranslational regulatory element.

The vectors in this toolkit are compatible with three-fragment Multi-Site Gateway cloning. In this system, specific DNA elements are first cloned into an “entry” vector flanked by unique attL and attR recombination sites. A “destination” vector is also required that contains an attR4/attR3-flanked negative-selection ccdB gene and chloramphenicol resistance cassette (ccdB/Cm^R^), which can contain additional vector-specific 5’ and 3’ flanking sequences. Next, using an “LR” reaction for site-specific recombination between pairs of unique attL and attR sites, DNA elements from a 5’, middle, and 3’ entry vector recombine to replace the destination vector ccdB/CmR cassette, allowing for the selection of recombinant clones (Fig 1A). Typically, the 5’ element contains a promoter sequence to drive gene expression, while middle and 3’ elements contain a gene of interest or other protein coding sequence.

The 5’ and 3’ sequences in the destination vector often dictate the application of the resulting construct. We created a destination vector with flanking sequences that allow for the production of lentivirus, which we call pEpic (Fig 1B). This configuration promotes viral RNA transcription in an antisense orientation to 5’ element-promoted gene expression. pEpic is a third-generation self-inactivating (SIN) vector [52, 53] with a 5’ Rev-response element (RRE), central polypurine tract (cPPT) and 3’ woodchuck hepatitis virus posttranscriptional regulatory element (WPRE) for enhanced viral titers and transgene expression [54–56]. pEpic also contains a dedicated antisense mouse phosphoglycerate kinase promoter (mPGK) driving expression of a puromycin resistance cassette (Puro^R^) for clonal selection purposes. Because large inserts between the viral long terminal repeats (LTRs) can decrease lentiviral titers [57, 58], we also generated pEpic without Puro^R^, which we call pEpic_Lite (Fig 1B). We designed pEpic and pEpic_Lite to produce antisense viral RNA because same-strand transcription of viral RNA has been shown to severely inhibit full-length viral RNA production and resulting viral titers [59].

A complete list of 5’ (p5E), middle (pME) and 3’ (p3E) entry vectors is provided in Table 1 and contains a short description of the vector, general use(s), use in figure(s), the constructing lab, and references to publications which previously used the vector. Below we provide examples of primary research applications of select vectors that highlight their diverse applications; each of which includes a schematic of the entry and destination vectors used in the LR reaction. In these schematics, bold vector names indicate novel vectors that are provided in the current toolkit. We conclude by outlining additional vectors provided in our toolkit and briefly discuss their potential applications.

**Table 1:** Entry vectors provided in the toolkit.

5' Entry Vectors	Description	Use(s)	Figure(s)	Lab	Reference
p5E-CMVmin	minimal cytomegalovirus immediate early enhancer/promoter (CMVmin) from pcDNA3	strong ubiquitous expression	3,4,5	Washbourne	
p5E-hPGK	human phosphoglycerate kinase (hPGK) promoter	ubiquitious expression	6	Stankunas	
p5E-EF1α/β-globin	frog translation elongation factor 1α (EF1α) enhancer fused to rabbit β-globin intron	semi-ubiquitous expression		Stankunas	[36]
p5E-EF1α/β-Actin	EF1α/β-globin fused to zebrafish β-actin 2 enhancer/promoter	strong semi-ubiquitous expression		Stankunas	
p5E-hSyn1	human Synapsin1 (hSyn1) promoter	pan-neuronal expression		Washbourne	
p5E-ESyn1	hSyn1 promoter fused to CMVmin	strong pan-neuronal expression		Washbourne	
p5E-*elavl3*	zebrafish *elavl3* promoter	pan-neuronal expression		Eisen	
p5E-*gfap*	zebrafish *gfap* promoter	glial-specific expression		Eisen	
p5E-*mnx1*	zebrafish *mnx1* promoter	expressed in primary and subset of secondary motorneurons, also expressed in VeLD		Eisen	[37]
p5E-*dusp6*	FGF-responsive regulatory element of the zebrafish *dusp6* promoter	FGF-responding cells		Stankunas	[38]
p5E-*krt5*	zebrafish *krt5* promoter	epidermis-specific expression		Stankunas	[36]
p5E-Ui4-eSIBR	human ubiquitin C promoter followed by intronically-expressed eSIBR cassette	ubiquitious expression, RNAi	3	Stankunas	
**Middle Entry Vectors**					
pME-eSIBR	intronically-expressed eSIBR cassette	RNAi	3	Washbourne	[30]
pME-tdTomato	orange-red fluorophore	imaging		Eisen	
pME-tdTomato no stop	tdTomato without stop codon	imaging, N-terminal conjugation		Eisen	
pME-mKate2 no stop	red fluorophore without stop codon	imaging, N-terminal conjugation		Washbourne	
pME-GFP-P2A	GFP without stop codon followed by porcine teschovirus-1 2A (P2A) "self-cleaving" peptide	imaging, bicistronic expression		Washbourne	
pME-nlsGFP-P2A	nuclear localization signal fused to N-terminus of GFP (nlsGFP) without stop codon, followed by P2A	nuclear-targeted GFP, imaging, bicistronic expression		Washbourne	
pME-memGFP-P2A	Fyn myristoylation domain fused to N-terminus of GFP (memGFP) without stop codon, followed by P2A	membrane-targeted GFP, imaging, bicistronic expression	5	Washbourne	
pME-BrainbowTEC	Brainbow-1.0 with fluorophores tdTomato-myc/EGFP/E2Crimson-HA	Cre-induced recombination for imaging, circuit tracing, cell mapping, lineage tracing	2	Eisen	
pME-FlEx switch	empty FlEx cassette containing two pairs of heterotypic, antiparallel *loxP*-type recombination sites	permits permenant, Cre-dependent inversion of inserted sequence		Eisen	
pME-ERT2-Cre-ERT2	tamoxifen-inducible estrogen receptor (ERT2) fused to both ends of Cre recombinase	tamoxifen-inducible Cre recombination		Stankunas	[38]
pME-GAL4-ERT2-VP16	GAL4 fused to ERT2 and the VP16 transcriptional activation domain	tamoxifen-inducible UAS-promoted transgene expression		Stankunas	[36]
**3' Entry Vectors**					
p3E-mKate2-HA no-pA	hemagluttanin (HA) epitope fused to C-terminus of mKate2 without pA	imaging, epitope labeling/purification		Washbourne	
p3E-mKate2-myc no-pA	myc epitope fused to C-terminus of mKate2 without pA	imaging, epitope labeling/purification		Washbourne	
p3E-GFP no-pA	GFP without pA	imaging		Washbourne	
p3E-nlsGFP pA	nlsGFP with pA	nuclear-targeted GFP, imaging	3	Washbourne	
p3E-nlsGFP no-pA	nlsGFP without pA	nuclear-targeted GFP, imaging	3	Washbourne	[30]
p3E-GFPmem pA	palmitoylation domain of human hRas fused to C-terminus of GFP (GFPmem) with pA	membrane-targeted GFP, imaging		Washbourne	
p3E-GFPmem no-pA	GFPmem without pA	membrane-targeted GFP, imaging		Washbourne	
p3E-CMVmin:GFP pA	CMVmin-promoted GFP with pA	imaging, bicistronic expression		Washbourne	
p3E-CMVmin:GFP no-pA	CMVmin-promoted GFP without pA	imaging, bicistronic expression		Washbourne	
p3E-CMVmin:nlsGFP pA	CMVmin-promoted nlsGFP with pA	nuclear-targeted GFP, imaging, bicistronic expression		Washbourne	
p3E-CMVmin:nlsGFP no-pA	CMVmin-promoted nlsGFP without pA	nuclear-targeted GFP, imaging, bicistronic expression		Washbourne	
p3E-CMVmin:GFPmem pA	CMVmin-promoted GFPmem with pA	membrane-targeted GFP, imaging, bicistronic expression		Washbourne	
p3E-CMVmin:GFPmem no-pA	CMVmin-promoted GFPmem without pA	membrane-targeted GFP, imaging, bicistronic expression		Washbourne	
p3E-GFP-HA no-pA	HA epitope fused to C-terminus of GFP without pA	imaging, epitope labeling/purification		Stankunas	
p3E-YFP-HA no-pA	HA epitope fused to C-terminus of YFP without pA	imaging, epitope labeling/purification		Stankunas	
p3E-CFP-HA no-pA	HA epitope fused to C-terminus of CFP without pA	imaging, epitope labeling/purification		Stankunas	
p3E-mCherry-HA no-pA	HA epitope fused to C-terminus of mCherry without pA	imaging, epitope labeling/purification		Stankunas	
p3E-HA no-pA	HA epitope without pA	epitope labeling/purification	3	Stankunas	
p3E-P2A-MCS no-pA	P2A followed by multiple cloning site without pA	bicistronic expression		Washbourne	
p3E-P2A-GFP no-pA	P2A followed by GFP without pA	imaging, bicistronic expression	4	Washbourne	
p3E-P2A-CFP no-pA	P2A followed by CFP without pA	imaging, bicistronic expression		Washbourne	
p3E-P2A-mKate2 no-pA	P2A followed by mKate2 without pA	imaging, bicistronic expression	4	Washbourne	
p3E-FRB-HA no-pA	HA epitope fused to C-terminus of TOR FKBP and rapamycin binding (FRB) domain without pA	Rapamycin/rapalog-induced dimerization (with FKBP)		Stankunas	
p3E-mCherry-FRB-HA no-pA	HA epitope fused to C-terminus and mCherry fused to N-terminus of FRB without pA	Rapamycin/rapalog-induced dimerization (with FKBP)	7	Stankunas	
p3E-FRB(KTF)-HA no-pA	HA epitope tag fused to C-terminus of FRB mutant (W2101F) without pA	Rapamycin/rapalog-induced dimerization (with FKBP)		Stankunas	
p3E-FRB(PLF)-HA no-pA	HA epitope tag fused to C-terminus of FRB mutant (K2095P, T2098L, W2101F) without pA	Rapamycin/rapalog-induced dimerization (with FKBP)		Stankunas	
p3E-FKBP-HA no-pA	HA epitope tag fused to C-terminus of human FK506 binding protein 12 (FKBP12) without pA	Rapamycin/rapalog-induced dimerization (with FRB)		Stankunas	
p3E-SGTAP no-pA	streptavidin binding protein (SBP) followed by a TEV cleavage site and 2 protein G copies without pA	tandem affinity protein purification	6	Stankunas	
p3E-Dam-myc no-pA	myc epitope tag fused to C-terminus of *E*. *coli* DNA adenine methyltransferase without pA	DamID for identifying native DNA binding sites of chromatin proteins		Stankunas	
p3E-*FRT*-Kan^R^-*FRT* pA	*FRT*-flanked kanamycin resistance cassette (Kan^R^) with pA	FLP-induced kanamycin resistance, drug selection		Stankunas	

### BrainbowTEC labels motoneuron circuitry of the developing zebrafish spinal cord

**Fig 2:**
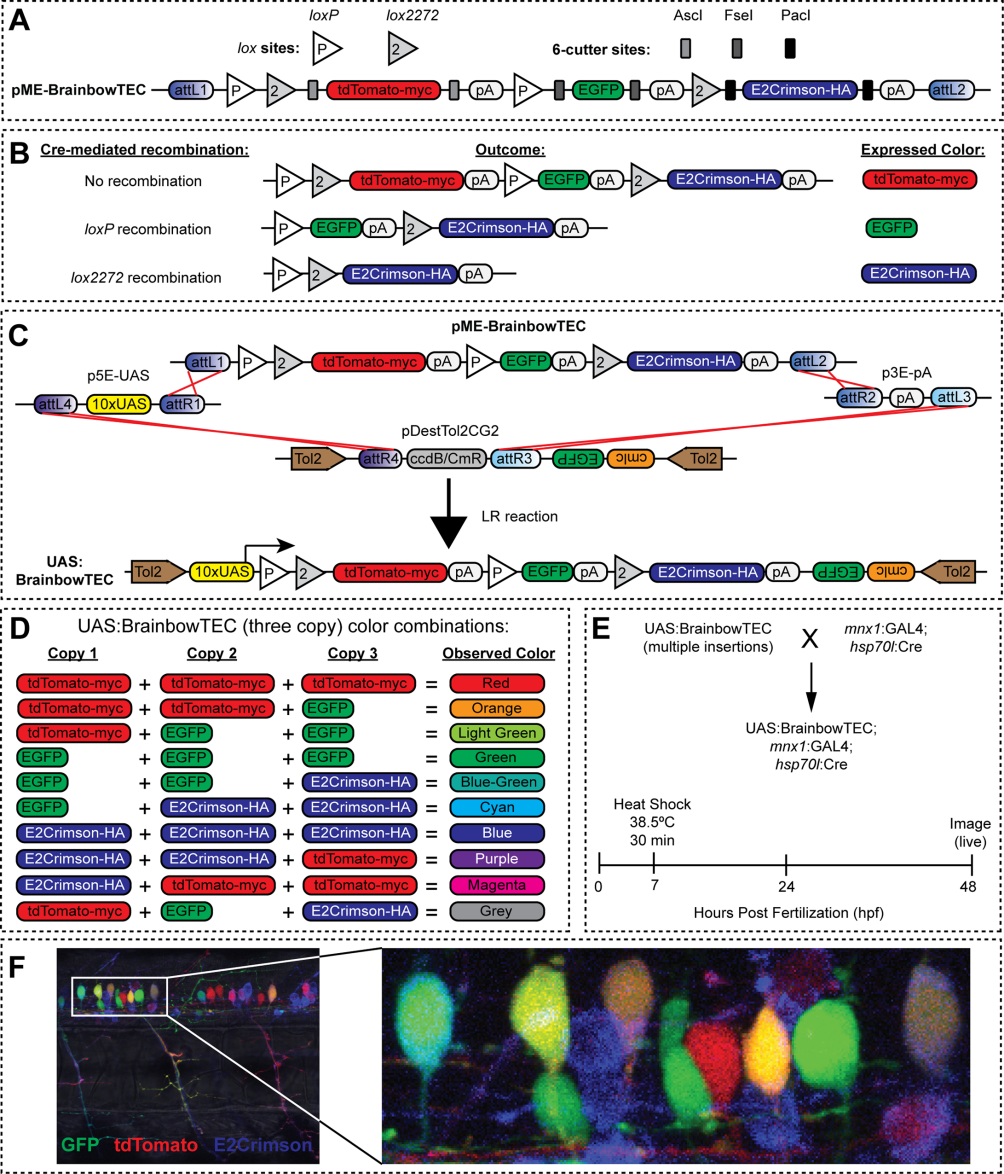
Overview of pME-BrainbowTEC and application in developing zebrafish spinal cord. (A) Schematic of pME-BrainbowTEC. (B) No recombination, *loxP* recombination or *lox2272* recombination lead to distinct fluorophore expression. (C) Schematic of LR recombination reaction used to create UAS:BrainbowTEC. (D) Possible fluorophore combinations and resulting observed color from three copy expression of UAS:BrainbowTEC. (E) Experimental design for UAS:BrainbowTEC labeling of motoneurons in developing zebrafish spinal cords. Fish carrying multiple copies of UAS:BrainbowTEC were crossed with a dual inducible-Cre and motoneuron-specific GAL4 driver line (*mnx1*:GAL4; *hsp70l*:Cre). Embryos were heat-shocked at 7 hours post fertilization (hpf) to induce Cre expression for *lox* recombination of genomic UAS:BrainbowTEC copies, then imaged at 48 hpf. (F) Representative fluorescent confocal microscope image for GFP, tdTomato, and E2Crimson in 48 hpf zebrafish embryo showing neuronal cell bodies in the spinal cord and motor axons within several adjacent somites. Inset shows neuronal cell bodies in the spinal cord.

To create transgenic brainbow zebrafish using the existing Gateway-compatible Tol2 transposon system [6], we developed pME-BrainbowTEC. This vector uses the brainbow-1.0 architecture [60], but with a novel color pallet comprised of a myc-tagged tdTomato, EGFP, and the far-red fluorophore E2Crimson [61] fused to a hemagglutinin (HA) epitope tag, which we abbreviate as TEC (Fig 2A). Brainbow has been revolutionary for the analysis of neural circuits, cell lineages, and tissue development [62]. The addition of epitope tags to tdTomato and E2-Crimson coupled with the existence of robust GFP antibodies makes BrainbowTEC compatible with immunolabeling; this is not possible with the original brainbow-1.0 system that utilized untagged GFP and XFP spectral variants [60]. The excitation maxima of these three fluorophores closely match the laser lines of commonly used three laser confocal imaging systems (488, 568 and 633nm), which is not the case for previous versions of Brainbow [63]. For ease of cloning different fluorophores in place of the current TEC combination, unique 6-cutter restriction enzyme sites were placed flanking each fluorophore (Fig 2A). The position of *loxP* and *lox2272* sites allows for either Cre-mediated recombination between different *lox* sites or failure to recombine dictating which fluorophore is expressed. tdTomato-myc is expressed as the default, no recombination configuration; whereas *loxP* recombination causes expression of GFP and *lox2272* recombination causes the expression of E2Crimson-HA (Fig 2B). For cell-specific brainbow expression, we performed an LR reaction with pME-BrainbowTEC and a 5’ element containing 10 copies of an upstream activating sequence (UAS) for the Gal4 transcriptional regulator into a destination vector for Tol2-mediated genomic integration [6], producing UAS:BrainbowTEC (Fig 2C). Three genomic copies of UAS:BrainbowTEC theoretically would allow 10 unique combinations of expressed fluorophores and resulting observed colors (Fig 2D).

We generated transgenic zebrafish founders containing multiple genomic integrations of BrainbowTEC. To specifically label motoneurons in embryonic zebrafish, we crossed UAS:BrainbowTEC fish with a driver line expressing GAL4 in primary motoneurons and a subset of secondary motoneurons [37] (*mnx1*:GAL4). This line also allowed temporal control of Cre expression by placing Cre downstream of the heat shock protein 70 promoter (*hsp70l*:Cre). The resulting embryos (UAS:BrainbowTEC; *mnx1*:GAL4; *hsp70l*:Cre) were heat shocked at 7 hours post fertilization (hpf) to induce Cre expression and recombination (Fig 2E). Live imaging of the embryos at 48 hpf showed robust BrainbowTEC labeling of motoneuron circuitry in the developing spinal cord (Fig 2F). Although we did not verify the number of insertions carried by individual embryos in the F1 generation, we validated that each reporter could be expressed after Cre-mediated recombination, and that embryos that carried multiple insertions gave rise to neurons that expressed multiple fluorescent reporters. These results show that BrainbowTEC is well-suited for circuit tracing in zebrafish *in vivo* and may be useful in other species and for other brainbow applications such as cell-lineage analysis.

### eSIBR-based lentiviral vectors enable potent multi-gene knockdown

amiRNAs are synthetic RNAi targeting sequences expressed from endogenous miRNA backbones, and have proven to be powerful tools for gene knockdown, especially when silencing of multiple genes is desired (for review see ref. [64]). In contrast to other common RNAi methods, such as RNA polymerase III-driven short hairpin RNAs (shRNAs), amiRNAs can be chained in tandem to target multiple genes and expressed from any RNA polymerase II-dependent promotor. This versatility in vector design allows amiRNAs to be co-expressed with protein coding sequences, such as fluorescent reporters. The two most commonly used amiRNA scaffolds are derived from human miR-30a [65] or from the mouse miR-155 (SIBR) [33] backbones. We have recently described an enhanced SIBR (eSIBR) backbone as an optimized amiRNA scaffold for potent knockdown of multiple targets from a single vector [30]. Here, we provide the eSIBR backbone in two vectors: p5E-Ui4-eSIBR and pME-eSIBR. p5E-Ui4-eSIBR drives amiRNA expression downstream of a dedicated hybrid human Ubiquitin C promoter (UbiC), whereas pME-eSIBR allows amiRNAs to be expressed from any promoter in a 5’ entry vector. In both vectors, eSIBR amiRNAs are expressed in an intron that is spliced from resulting mRNAs, which prevents amiRNA cleavage from inhibiting downstream transgene expression and also increases amiRNA knockdown potency compared to exonically-expressed amiRNAs [30, 33].

Lentivirus provide a means for gene transfer into many cell types which are not amenable to transgenesis by other methods, including neurons, and are therefore a useful approach for introducing amiRNAs. Because transgenes carried by lentivirus are genomically integrated, they are beneficial for many research applications such as clonal selection and long-term expression. As with other expression constructs, promoter selection and inclusion of other factors such as polyadenylation signal (pA) sequences can influence the efficacy of gene expression from lentiviral vectors. Additionally, high viral titers are often necessary for efficient transgenesis in certain applications, such as *in vivo* injections. Therefore, we investigated these factors in an attempt to optimize eSIBR-based gene knockdown and viral production from our pEpic destination vectors.

**Fig 3:**
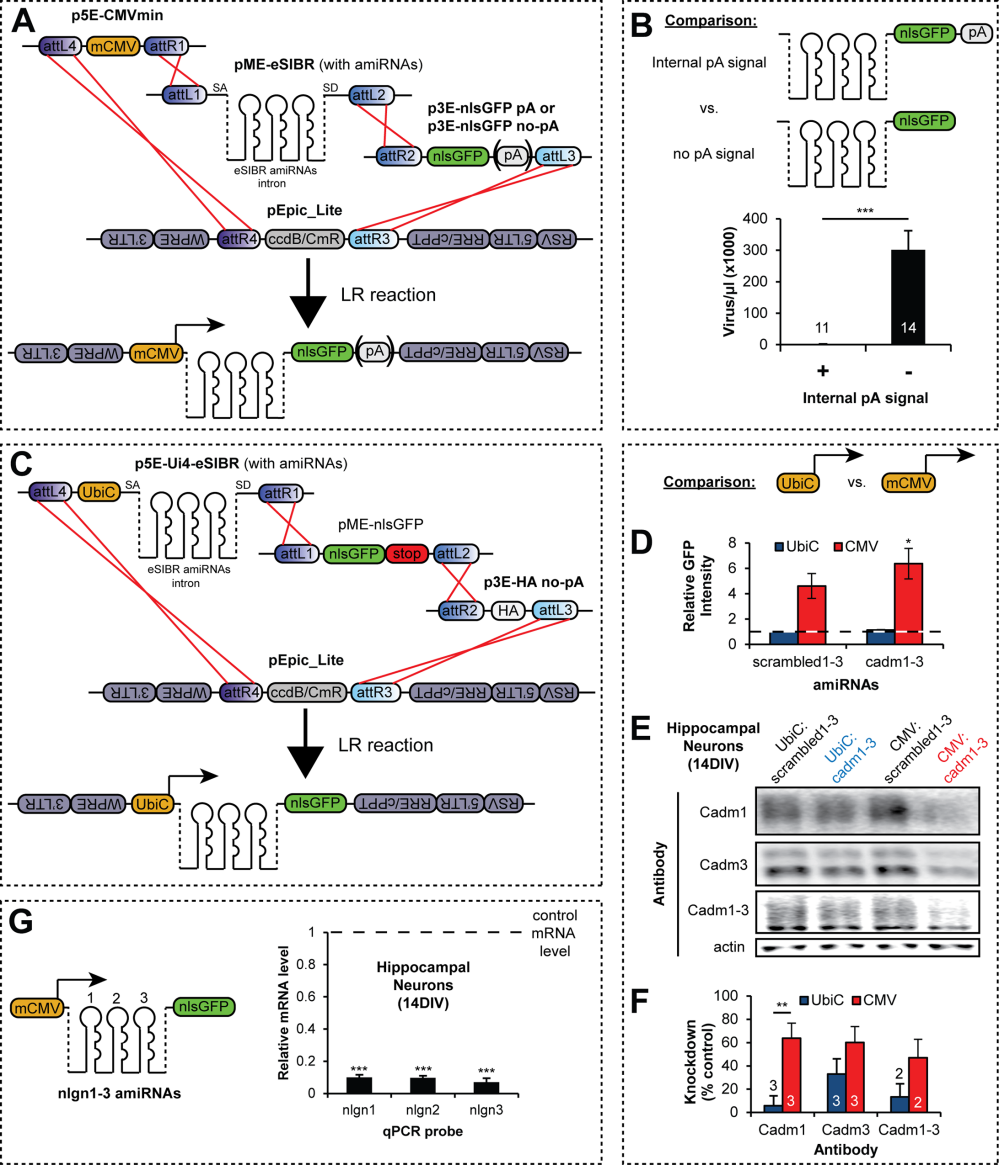
Creation and optimization of potent multi-target lentiviral knockdown constructs using eSIBR-based artificial miRNA vectors. (A) Schematic of LR recombination reactions used to create pEpic_Lite mCMV:eSIBR-nlsGFP pA and no-pA vectors. (B) Lentiviral titers obtained from mCMV:eSIBR-nlsGFP vectors with or without a pA signal sequence. Number of biological replicates (n) are shown on or above the bars. (C) Schematic of LR recombination reaction used to create pEpic_Lite UbiC:eSIBR-nlsGFP no-pA vectors. pME-nlsGFP contains a stop codon so the HA epitope in p3E-HA no-pA is not expressed. (D) Mean GFP intensity as measured by flow cytometry of HEK293T cells infected at single-copy levels with UbiC or CMVmin-promoted eSIBR vectors carrying scrambled1-3 or cadm1-3 amiRNAs. For scrambled1-3 and cadm1-3 groups, GFP intensity was set relative to UbiC-promoted GFP levels at an arbitrary value of 1 (dashed line). n = 3 biological replicates. (E) Representative quantitative western blots for antibodies against Cadm1, Cadm3, or Cadm1-3 and (F) quantification of protein knockdown from 14DIV cultured hippocampal neurons infected with lentivirus carrying amiRNAs against cadm1-3 compared to corresponding scrambled1-3 amiRNA infected control neurons. Number of biological replicates (n) is noted on or above bars. (G) Quantification of nlgn1, 2 and 3 mRNA levels by qRT-PCR from 14DIV cultured hippocampal neurons infected at saturating levels with lentivirus carrying a pEpic_Lite mCMV:eSIBR no-pA vector with amiRNAs against nlgn1, 2 and 3. mRNA levels were set relative to control sister cultures infected with a construct carrying scrambled1-3 amiRNAs (dashed line). (B,D,F,G) *p<0.05, **p<0.01 and ***p<0.001; Student’s two-tailed t-tests.

Because previous reports have observed that internal pA sequences can impact lentiviral production [59, 66], we wanted to determine if the inclusion of a pA sequence in pEpic-based lentiviral constructs affected the resulting titer. We inserted previously described chained eSIBR amiRNAs targeting synaptic cell adhesion molecule family members (cadm1-3, nlgn1-3, or nrxn1-3) or scrambled amiRNA sequences targeting no known genes (scrambled1-3) into pME-eSIBR [30]. Next, we recombined pME-eSIBR amiRNAs with a 5’ entry vector containing the minimal cytomegalovirus immediate early enhancer/promoter (p5E-CMVmin) and a 3’ entry vector encoding a nuclear-localized GFP (p3E-nlsGFP) with or without a pA sequence into the destination vector pEpic_Lite to create mCMV:eSIBR-nlsGFP pA and no-pA vectors (Fig 3A). Finally, we produced lentivirus with these vectors in HEK293T cells. Strikingly, the presence of an internal pA signal in the lentiviral vector reduced the resulting titer >200-fold (Fig 3B). Lentiviral vectors without a pA sequence, however, still produced robust transgene expression (data not shown). Therefore, we developed numerous 3’ entry vectors without pA sequences (Table 1) and strongly recommend using only 3’ entry vectors lacking a pA sequence for production of lentivirus.

To compare the effect of promoter choice on knockdown potency, we cloned cadm1-3 and scrambled1-3 eSIBR amiRNAs into p5E-Ui4-eSIBR. We then performed LR reactions using p5E-Ui4-eSIBR constructs with a middle entry vector carrying nlsGFP with a stop codon (pME-nlsGFP [6]) and our smallest no-pA 3’ entry vector (p3E-HA no-pA) and pEpic_Lite to create UbiC:eSIBR-nlsGFP no-pA vectors (Fig 3C). Because the nlsGFP contained a stop codon, the HA tag from p3E-HA no-pA was not expressed, and the vector functioned as a “filler” to allow the LR reaction to occur. Next, we produced lentivirus carrying these constructs and infected HEK293T cells with single lentiviral particles, and then assayed nlsGFP expression intensity in transduced cells by flow cytometry. For lentiviral vectors carrying cadm1-3 or scrambled1-3 amiRNAs, the mean GFP fluorescence intensity was >4-fold higher from the CMVmin promoter than from the UbiC promoter (scrambled amiRNAs p = 0.07, cadm1-3 amiRNAs p<0.05, Student’s two-tailed t-tests, Fig 3D). To determine if enhanced expression from the CMVmin promoter could increase knockdown efficiency, we transduced primary cultured rat hippocampal neurons with sub-saturating concentrations of lentivirus carrying cadm1-3 or scrambled1-3 amiRNAs expressed from either the UbiC or CMVmin promoter. Quantitative western blot analysis using antibodies for Cadm1 or Cadm3 or an antibody that binds Cadm1-3 showed in each case that knockdown potency was enhanced when amiRNAs were expressed from the CMVmin promoter compared to UbiC (Fig 3E & 3F; Cadm1 p<0.01, Cadm3 p = 0.11, Cadm1-3 p = 0.11, Student’s two-tailed t-test). Together, these results highlight the importance of promoter choice for optimizing knockdown from eSIBR amiRNAs.

We previously showed that chained eSIBR amiRNAs expressed from lentiviral vectors potently knocked down Cadm1-3 in cultured rat hippocampal neurons when cells were infected at saturating titers [30]. To determine if eSIBR amiRNAs produced efficient knockdown of other genes, we similarly infected cultured neurons at saturating titers with lentivirus carrying eSIBR amiRNAs against nlgn1-3. qRT-PCR analysis showed that mRNA levels for each nlgn gene were reduced >90% compared to control cultures infected with scrambled1-3 amiRNAs (Fig 3G). These observations provide more evidence that the eSIBR backbone is a potent tool for multi-gene knockdown.

Prior Gateway toolkits have provided entry vectors containing miR-30a scaffolds for amiRNA expression [12, 67]. However, to our knowledge, only one report provides Gateway-compatible vectors with the SIBR/mouse miR-155-based amiRNA backbone [68]. Intriguingly, this study showed that SIBR-based amiRNAs vastly outperformed other amiRNA scaffolds, including miR-30. Further, this study was the first to demonstrate efficient, heritable knockdown in zebrafish from an RNAi-based method. Because our toolkit vectors were specifically developed to be compatible with the Tol2kit [6], a popular Gateway vector collection for the rapid generation of transgenic zebrafish lines, the eSIBR vectors described here may potentially represent a powerful loss-of-function tool for the zebrafish research community. We have not yet attempted the use of eSIBR-based amiRNAs for gene knockdown in zebrafish and therefore cannot comment on its efficacy in this organism. Therefore, optimization will likely need to occur before pioneering the use of eSIBR-based amiRNAs in zebrafish.

### P2A vectors provide high-fidelity bicistronic transmembrane protein expression

Despite the rapid advancement in recombinant DNA strategies, expression of more than one transgene from a single vector often remains challenging. A common method for dual-gene expression includes use of the internal ribosome entry site (IRES) between two protein coding sequences; however, IRES is notoriously inefficient and expression of the downstream transgene tends to be greatly diminished [69, 70]. Another method for two-protein expression is to use a dedicated promoter for each protein coding sequence [69, 71–73]. Unfortunately, limited understanding about gene expression from independent promoters in close proximity to each other has prevented the development of an effective, universal dual-promoter system that works in all vector systems. For example, depending upon the application, independently promoted genes placed in tandem on the same strand can cause unpredictable promoter suppression or transcriptional interference, which is especially prevalent in retroviral and lentiviral vectors [74–77].

Recently, viral 2A peptides have been extremely successful for stoichiometric expression of two proteins from a single open reading frame (ORF) in recombinant DNA vectors [42, 78–82]. Initially, the mechanism of 2A bicistronic expression was thought to be mediated by proteolytic cleavage of the growing polypeptide chain, and was therefore touted as “self-cleaving” [83]; further investigations instead suggested a translational “ribosomal-skip” event leads to the production of two independent protein products when 2A peptides were placed between two protein coding sequences [84]. Of the various 2A and 2A-like peptides, the P2A sequence has been most commonly and successfully applied for one-to-one protein expression in a wide range of cell types and organisms [31, 42]. Therefore, we developed several P2A 3’ entry vectors without a pA signal for independent gene expression from a protein-of-interest’s C-terminus. In these constructs, GFP, CFP or the bright red fluorophore mKate2 [39] were placed downstream of P2A. Additionally, we made a vector with a multi-cloning site (MCS) downstream of P2A for inserting other protein sequences.

**Fig 4:**
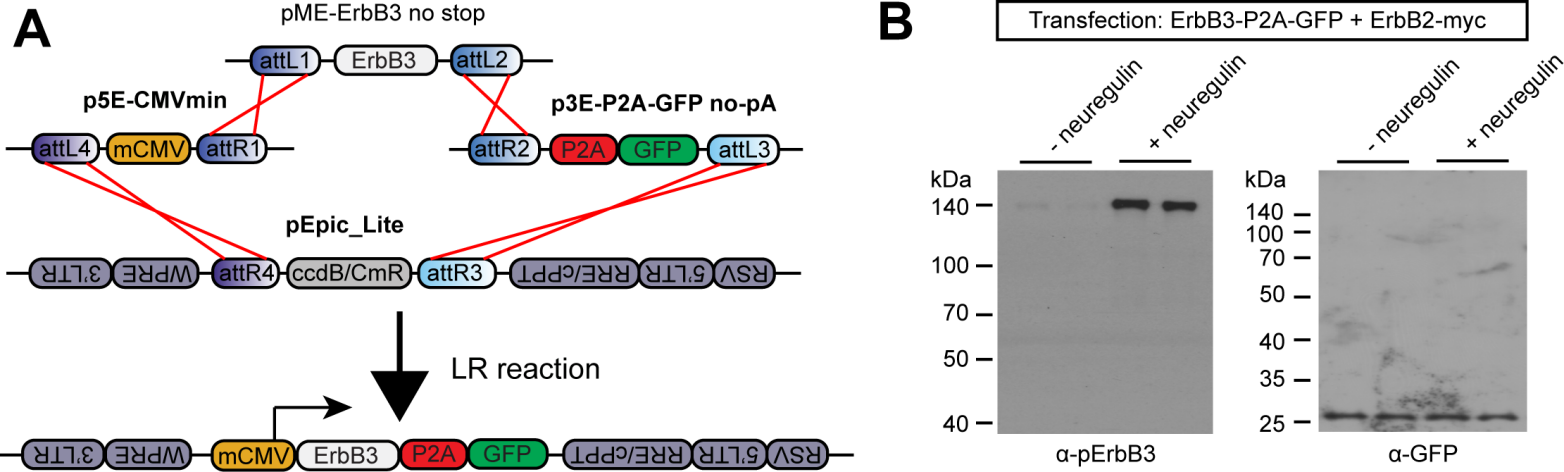
Efficient bicistronic expression from C-terminal P2A conjugation. (A) Schematic of LR recombination reaction used to create pEpic_Lite mCMV:ErbB3-P2A-GFP. (B) Western blots of COS7 cell lysates 48 hours after co-transfection with ErbB3-P2A-GFP and ErbB2-myc, with or without treatment with neuregulin. Immunoblotting was performed with antibodies against phosphorylated ErbB3 (pErbB3) or GFP.

To validate the efficacy of p3E-P2A vectors for bicistronic fluorophore expression, we generated a plasmid driving expression of the receptor tyrosine kinase ErbB3 conjugated to P2A-GFP to make mCMV:ErbB3-P2A-GFP (Fig 4A). ErbB3, a transmembrane protein, is a member of the epidermal growth factor receptor family and is a receptor for the growth factor neuregulin/heregulin [85]. However, ErbB3 itself is incapable of kinase activity and signal transduction [86]; instead it forms heterodimers with and is phosphorylated by ErbB2 following neuregulin binding [87, 88]. To determine if conjugation of P2A-GFP to the C-terminus of ErbB3 impacts phosphorylation by ErbB2, we co-transfected COS7 cells with mCMV:ErbB3-P2A-GFP and a construct driving expression of a myc-tagged ErbB2 (ErbB2-myc). 48 hours after transfection we briefly treated the cells with neuregulin1-β to induce phosphorylation by ErbB2. Western blotting for phosphorylated ErbB3 (pErbB3) showed that the addition of P2A-GFP did not inhibit phosphorylation (Fig 4B). Further, because ErbB3 is a transmembrane protein, this observation shows that the P2A sequence did not hinder correct plasma membrane targeting. Lastly, to determine the reliability of stoichiometric ErbB3 and GFP protein expression from this construct, we also performed western blotting for GFP on the same samples. Blots showed that GFP was only present at its predicted molecular weight (~27 kDa), but not at the size of ErbB3 (~140 kDa) (Fig 4D), suggesting high-fidelity 2A “cleavage.” Although others have reported inhibition of “cleavage” when other 2A sequences were placed downstream of a protein containing an N-terminal signal sequence [89, 90], we did not observe this problem. This result demonstrates that our P2A 3’ entry vectors can be effectively used with proteins bearing an N-terminal signal sequence, such as transmembrane and secreted proteins.

**Fig 5:**
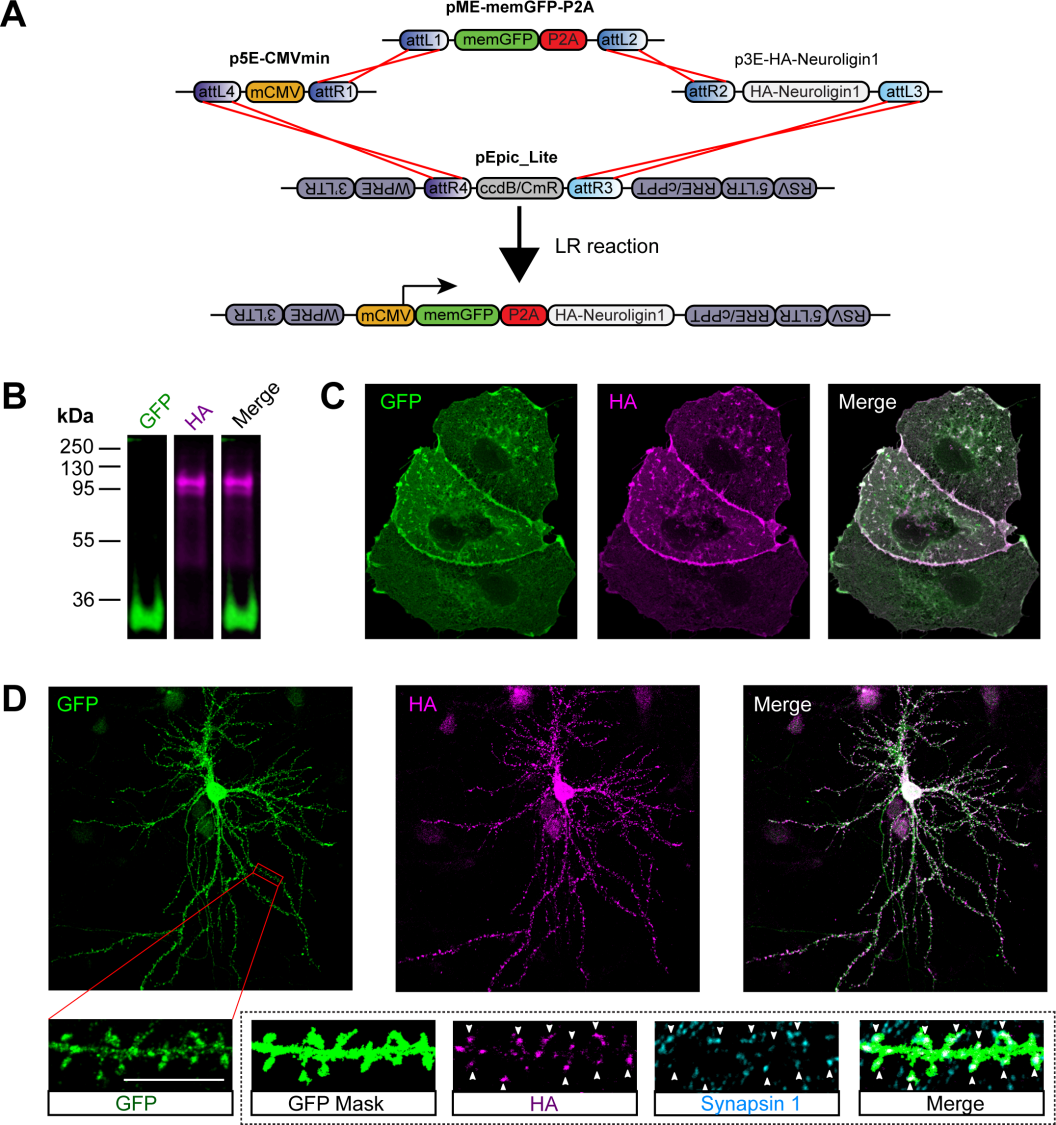
Effective dual protein expression through N-terminal P2A conjugation to HA-Neuroligin1. (A) Schematic of LR recombination reaction used to create pEpic_Lite mCMV:memGFP-P2A-HA-Neuroligin1. (B) Dual fluorescent western blot of COS7 cell lysate 24 hours after transfection with mCMV:memGFP-P2A-HA-Neuroligin1. Immunoblotting was performed with antibodies against GFP and HA. (C) Immunocytochemistry for GFP and HA in COS7 cells 24 hours after transfection with mCMV:memGFP-P2A-HA-Neuroligin1. Cells were fixed with paraformaldehyde and surface stained for HA, then permeabilized and stained for GFP. (D) Immunocytochemistry for GFP, HA and the synaptic vesicle-associated protein Synapsin1 in cultured rat hippocampal neurons. Cells were transduced with lentivirus carrying mCMV:memGFP-P2A-HA-Neuroligin1at 2DIV and fixed for immunolabeling at 14DIV. Cells were surface stained for HA, then permeabilized and stained for GFP and Synapsin1. Inset is of an individual basal dendrite segment; the GFP mask is a binarized image of the dendrite using intensity thresholding of the GFP signal. Arrowheads mark dendritic spines containing HA and co-localized Synapsin1 puncta. Scale bar = 10 μm.

Because “cleavage” of the 2A peptide leaves the majority of the P2A sequence attached to the C-terminus of the upstream protein, p3E-P2A constructs are not suitable for bicistronic expression of proteins where addition of a C-terminal peptide would compromise protein function. For example, many cell adhesion molecules, including the neuroligin family, present a PDZ-binding motif in their terminal 4 amino acids [91]; the addition of a protein tag to the C-terminus would prevent their association with crucial PDZ domain-containing scaffolding proteins. Also, it was important to investigate whether placement of a P2A peptide upstream of an endoplasmic reticulum (ER) signal sequence would result in correct targeting of a transmembrane protein at the plasma membrane. We developed P2A middle-entry vectors for N-terminal bicistronic expression of GFP, nlsGFP and a GFP that targets to the plasma membrane (memGFP) by the addition of the Fyn myristoylation domain to its N-terminus [41]. To validate this approach, we generated an expression vector with memGFP-P2A conjugated to HA-Neuroligin1 (mCMV:memGFP-P2A-HA-Neuroligin1, Fig 5A). Dual-color western blotting for GFP and the HA-tag again showed high-fidelity P2A-mediated “cleavage” of the construct into discrete proteins when expressed in COS7 cells (Fig 5B). Further, HA immunolabeling of non-permeabilized cells showed that HA-Neuroligin1 was correctly expressed on the cell surface (Fig 5C). These results demonstrate that our P2A middle-entry vectors can be effectively utilized for stoichiometric polyprotein expression, and that N-terminal P2A conjugation does not disrupt proper subcellular targeting of downstream proteins bearing a signal sequence.

We additionally determined if this vector worked for bicistronic expression in cultured rat hippocampal neurons. Again, surface labeling for HA in neurons transduced with lentivirus carrying mCMV:memGFP-P2A-HA-Neuroligin1 showed that Neuroligin1 was correctly inserted into the plasma membrane (Fig 5D). Further, co-labeling experiments demonstrated that memGFP was robustly expressed in transduced neurons, and that postsynaptic HA-Neuroligin1 was highly co-localized with presynaptic puncta marked by the synaptic vesicle-associated protein Synapsin1 along dendrites in these cells (Fig 5D). We conclude that HA-Neuroligin1 expressed from this vector retained its proper synaptic targeting, demonstrating the utility of our P2A constructs for bicistronic gene expression in primary neurons. Collectively, these results show our P2A vectors are efficient, useful tools for bicistronic expression of proteins, including transmembrane proteins.

### Efficient protein isolation using tandem affinity purification

**Fig 6:**
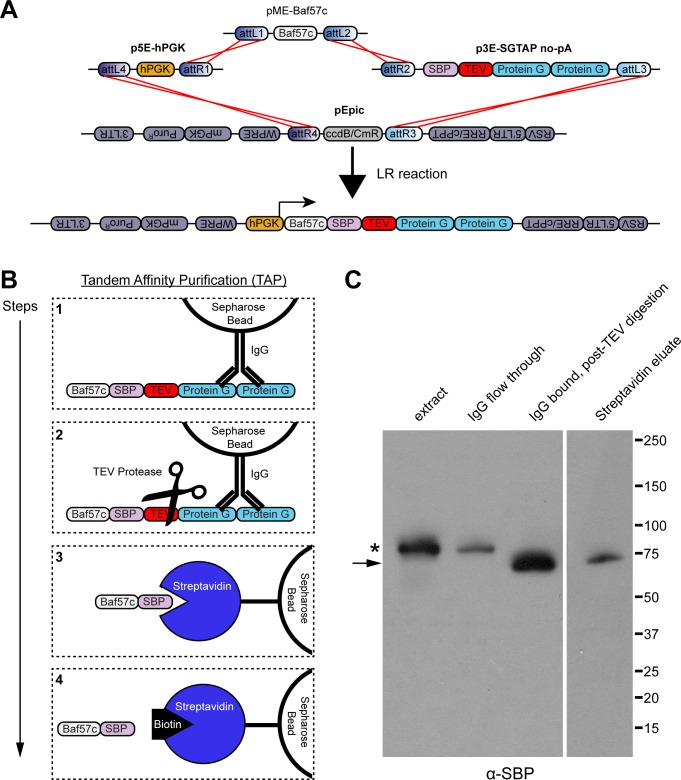
Tandem affinity purification of chromatin-remodeling complex protein Baf57c using SGTAP. (A) Schematic of LR recombination reaction used to create pEpic CMV:Baf57c-SGTAP. (B) Schematic of steps for TAP of Baf57-SGTAP. Step 1: Baf57c with a C-terminally conjugated SBP, TEV protease cleavage site, and tandem copies of protein G is first isolated by affinity purification using IgG-sepharose beads; Step 2: Baf57c-SBP is cleaved from protein G bound to IgG-sepharose beads by the addition of TEV protease; Step 3: Baf57c-SBP is further isolated by affinity purification using streptavidin-sepharose beads; Step 4: Baf57c-SBP is finally eluted from streptavidin by the addition of biotin. (C) Western blot for SBP at various stages of Baf57c purification from nuclear extracts of HEK293T cells expressing pEpic CMV-Baf57c-SGTAP. 10% of each indicated fraction was used for immunoblotting. Lane 1: the crude nuclear extract; Lane 2: nuclear extract after incubation with IgG beads; Lane 3: post-TEV protease cleavage of proteins bound to IgG beads; Lane 4: SDS elution of proteins from beads following Streptavidin purification. The asterisk indicates Baf57c-SGTAP fusion proteins; the arrow indicates the cleaved Baf57c-SBP fusion; molecular weights in kilodaltons are shown at the right.

Affinity purification has been instrumental in determining protein complex compositions; however, pull-downs from fusion proteins with a single affinity tag, such as Protein A or Protein G, are often impure and may lead to false-positive identification of protein complex members. Protein purification using sequential isolations from two different affinity tags considerably reduces non-specific protein pulldowns, but is notorious for low overall yield. We developed a 3’ entry vector, p3E-SGTAP (streptavidin binding protein/Protein G tandem affinity purification), based on an optimized tandem affinity purification (TAP) method that is amenable to much smaller amounts of starting material [44]. To validate the use of this vector for TAP, we generated a construct with our 5’ entry vector containing a human PGK promoter (p5E-hPGK) driving expression of the chromatin remodeling-complex protein Baf57c/Smarce1 [92] fused to SGTAP fusion protein in our pEpic lentiviral destination vector (hPGK:Baf57c-SGTAP, Fig 6A). Purification of SGTAP fusion proteins involves a first round of Protein G affinity purification by IgG-bead pulldowns, on-bead cleavage of the fusion protein by TEV protease, a second round of streptavidin binding protein (SBP) affinity purification by streptavidin-bead pulldowns, and elution of the SBP-protein from streptavidin by the addition of biotin (Fig 6B). To determine the efficacy of this procedure for isolating Baf57c-SGTAP, we infected HEK293T cells with lentivirus carrying the CMV:Baf57c-SGTAP construct and selected for transduced cells with puromycin. TAP was performed on cell lysates, which showed that sufficient amounts of protein were isolated to use for analyzing protein complex interactions (Fig 6C). These results suggest that p3E-SGTAP can be effectively utilized for affinity purification.

### Use of rapamycin-induced dimerization to force nuclear export

Manipulating protein activity using highly specific small molecules allows the rapid and reversible control of gene/protein function. A parallel chemical genetics effort uses “bi-functional” molecules that simultaneously bind to two specific protein domains. The two domains can be fused to target proteins (or functional protein motifs) to enable their chemically-inducible dimerization (CID). CID can be widely applied, for example, for inducible membrane recruitment, nuclear import/export, and protein degradation [93]. One exemplary CID system uses the macrolide rapamycin or its engineered derivatives called “rapalogs” [45, 47]. Rapamycin, an anti-fungal antibiotic from *Streptomyces hygroscopicus* [94], forms a ternary complex with a FK506 binding protein (FKBP)-tagged and FKBP-rapamycin binding (FRB) domain-tagged protein [95, 96]. We developed a line of 3’ entry vectors for the generation of FRB or FKBP fusion proteins for rapamycin-induced dimerization (p3E-FRB-HA no-pA, p3E-mCherry-FRB-HA no-pA, and p3E-FKBP-HA no-pA). We also generated a vector containing an engineered version of the FRB domain, p3E-FRB(KTF)-HA no-pA, that has selective affinity for MaRap, a detoxified rapalog [45]. Further, we provide p3E-FRB(PLF)-HA no-pA, which encodes an FRB domain mutant that highly destabilizes fusion proteins [46]. Protein degradation from FRB(PLF) fusion can be prevented by the addition of rapamycin or MaRap; therefore, this construct can be used for “inducible stabilization” [46].

**Fig 7:**
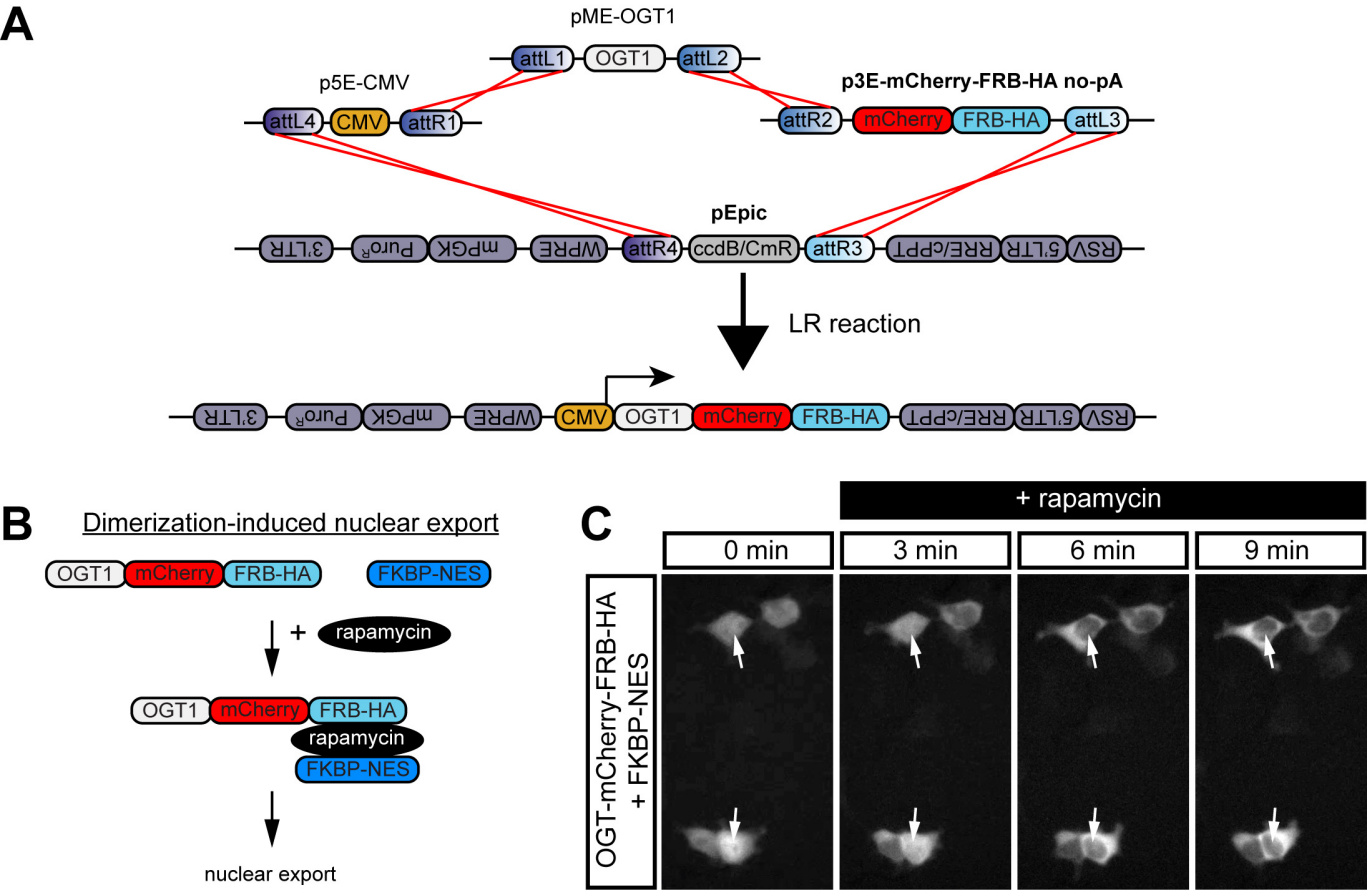
Rapamycin-induced dimerization to drive nuclear export. (A) Schematic of LR recombination reaction used to create pEpic CMV:OGT1-mCherry-FRB-HA. (B) Schematic of rapamycin-induced dimerization with FKBP-NES to drive nuclear export. (C) Time-lapse imaging (S1 Movie) of mCherry in HEK293T cells transfected with pEpic CMV:OGT1-mCherry-FRB-HA and FKBP-NES. Rapamycin was added at time 0. White arrows mark cell nuclei.

The FRB-rapamycin-FKBP system has been widely applied including to force protein subcellular localization and to modulate cellular signaling [93, 97]. To demonstrate the utility of our vectors for CID, we created an expression vector for O-GlcNAc transferase (OGT1), an enzyme implicated in Polycomb group chromatin regulation and other nuclear functions [98], fused to FRB-mCherry-HA (CMV:OGT1-mCherry-FRB-HA, Fig 7A). To inhibit OGT1 function, we applied a method to force nuclear export of FRB-tagged OGT1 by rapamycin-induced dimer formation with a nuclear export sequence fused to FKBP (FKBP-NES) (Fig 7B). Using live imaging, we observed that the addition of rapamycin to HEK293T cells co-transfected with OGT1-mCherry-FRB-HA and FKBP-NES caused rapid nuclear export of OGT1 (Fig 7C and S1 Movie). This result highlights a primary application of CID using constructs derived from our FRB and FKBP entry vectors.

### Additional entry vectors

#### 5’ entry vectors

We generated additional 5’ entry vectors containing promoters for ubiquitous, semi-ubiquitous, or cell/tissue-specific gene expression. These include two promoters for ubiquitous or semi-ubiquitous expression: frog elongation factor 1α fused to rabbit β-globin intron (p5E-EF1α/β-globin) or EF1α/β-globin additionally enhanced with a zebrafish β-actin 2 promoter [36] (p5E-EF1α/β-Actin). We provide three promoters for pan-neuronal expression: human synapsin1 promoter alone (p5E-hSyn1) or enhanced CMVmin hybrid promoter [99] (p5E-ESyn1) and the zebrafish neuron-specific RNA-binding protein *elavl3* [100] (p5E-*elavl3*). We offer four additional zebrafish gene promoters for cell/tissue-specific expression in glia [35] (p5E-*gfap*), primary motoneurons [37] (p5E-*mnx1*), epidermis [36] (p5E-*krt5*), and FGF-responsive cells [38, 101, 102] (p5E-*dusp6*).

#### Middle entry vectors

For imaging, we created an additional middle entry vector with the orange-red fluorophore tdTomato [40] with a stop codon (pME-tdTomato). For N-terminal protein fusions, we made vectors with tdTomato and mKate2 without stop codons (pME-tdTomato no stop and pME-mKate2 no stop).

We also created a vector containing an empty FlEx switch cassette (pME-FlEx switch), which allows the insertion of a protein coding sequence between two pairs of heterotypic, antiparallel *loxP*-type recombination sites [103, 104]. Cre-mediated recombination causes the inversion of the coding sequence and excision of two recombination sites, which inhibits further recombination. Therefore, if the coding sequence is inserted into pME-FlEx switch in an antisense direction, Cre-mediated inversion results in persistent expression, as long as the switch is placed downstream of an active promoter. Conversely, if the coding sequence is inserted in the sense orientation, Cre-mediated recombination will result in permanently extinguishing expression. Further, if two protein-coding sequences are inserted, one in the sense and the other in the antisense orientation, Cre-mediated inversion will turn “on” the antisense protein and turn “off” the sense protein, which allows monitoring of Cre activity *in vivo* [104, 105].

Finally, we offer vectors for tamoxifen-inducible gene expression by linking the estrogen receptor variant ERT2 onto both ends of Cre (pME-ERT2-Cre-ERT2) which can be used for *lox* site recombination. pME-ERT2-Cre-ERT2 differs from a previously described Cre-ERT2 middle entry vector [106] in that it contains a second copy of ERT2 on the N-terminus of Cre. In zebrafish, this abolished high background recombination activity observed with Cre fused to a single ERT2 copy in the absence of tamoxifen and allowed tight regulation of transgene expression [107]. ERT2 was also fused to GAL4 and the VP16 transcriptional activation domain (pME-GAL4-ERT2-VP16) for expression of UAS-linked transgenes. Both of these vectors have been successfully used to generate conditional transgenic zebrafish lines with temporally controlled gene expression [36, 38].

#### 3’ entry vectors

We developed additional 3’ entry vectors for generating C-terminal fusion proteins. This list includes a no-pA vector with GFP (p3E-GFP no-pA) and pA and no-pA vectors with a GFP that targets to the plasma membrane (GFPmem) via the addition of an hRas palmitoylation domain on its C-terminus (p3E-GFPmem pA and no-pA). We stress the distinction of GFPmem in the 3’ entry vectors from memGFP in pME-memGFP-P2A because they use distinct mechanisms to localize at the plasma membrane. We also provide no-pA vectors containing mKate2 with myc or HA tags (p3E-mKate2-myc and p3E-mKate2-HA no-pA). Additionally, we generated five no-pA vectors for generating C-terminal HA-tagged fluorophore fusion proteins (p3E-GFP-HA no-pA, p3E-YFP-HA no-pA, p3E-CFP-HA no-pA and p3E-mCherry-HA no-pA). We also cloned a myc-tagged *E*. *coli* DNA adenine methyltransferase (p3E-Dam-myc no-pA), which, when fused to a chromatin-associated protein of interest, can be used to identify DNA binding sequences [43, 108]. Lastly, we provide a pA vector containing a *FRT*-flanked kanamycin resistance cassette (p3E-*FRT*-Kan^R^-*FRT* pA) for FLP-induced antibiotic resistance.

We also developed 3’ entry vectors for two-gene expression constructs from independent promoters. Using a dedicated CMVmin promoter, we made both pA and no-pA vectors for independent expression of GFP, nlsGFP or GFPmem (p3E-CMVmin:GFP pA and no-pA, p3E-CMVmin:nlsGFP pA and no-pA, and p3E-GFPmem pA and no-pA). However, we warn against using these vectors to generate bicistronic constructs if robust expression of an upstream transgene is required because, as with other reports [76, 77, 109], we have observed that expression of transgenes placed 5’ of CMVmin:GFP inserts were severely reduced (data not shown).

### Concluding Remarks

Multisite Gateway cloning is a powerful method for generating multi-component vectors. Collectively, we have developed more than 50 novel entry vectors and two lentiviral destination vectors for vertebrate expression constructs. These vectors are available, either individually or as an entire kit, via Addgene. Our constructs complement existing vertebrate-compatible Gateway toolkits, including the widely-used Tol2kit [6]. Further, our vectors may be utilized for genome-wide applications, such as high-throughput screens, when coupled to large Gateway-based ORFeome collections, including the near-complete human ORFeome V8.1[22] that is now available from the DNASU repository [110] (www.dnasu.org). Therefore, this toolkit should prove useful for rapidly generating constructs that will facilitate molecular and cellular research.

## Supporting Information

S1 Movie Time-lapse video of rapamycin-induced nuclear export. (AVI)
