# Correction: A MultiSite Gateway Toolkit for Rapid Cloning of Vertebrate Expression Constructs with Diverse Research Applications

**DOI:** 10.1371/journal.pone.0176543

**Published:** 2017-04-20

**Authors:** Daniel K. Fowler, Scott Stewart, Steve Seredick, Judith S. Eisen, Kryn Stankunas, Philip Washbourne

In Fig 1, the orientation of vector-specific 5’ and 3’ sequences for pEpic and pEpic_Lite and derivatives of these vectors are incorrect. The correct configurations are all sense-strand oriented components. Please see the corrected Fig 1 here.

**Fig 1:**
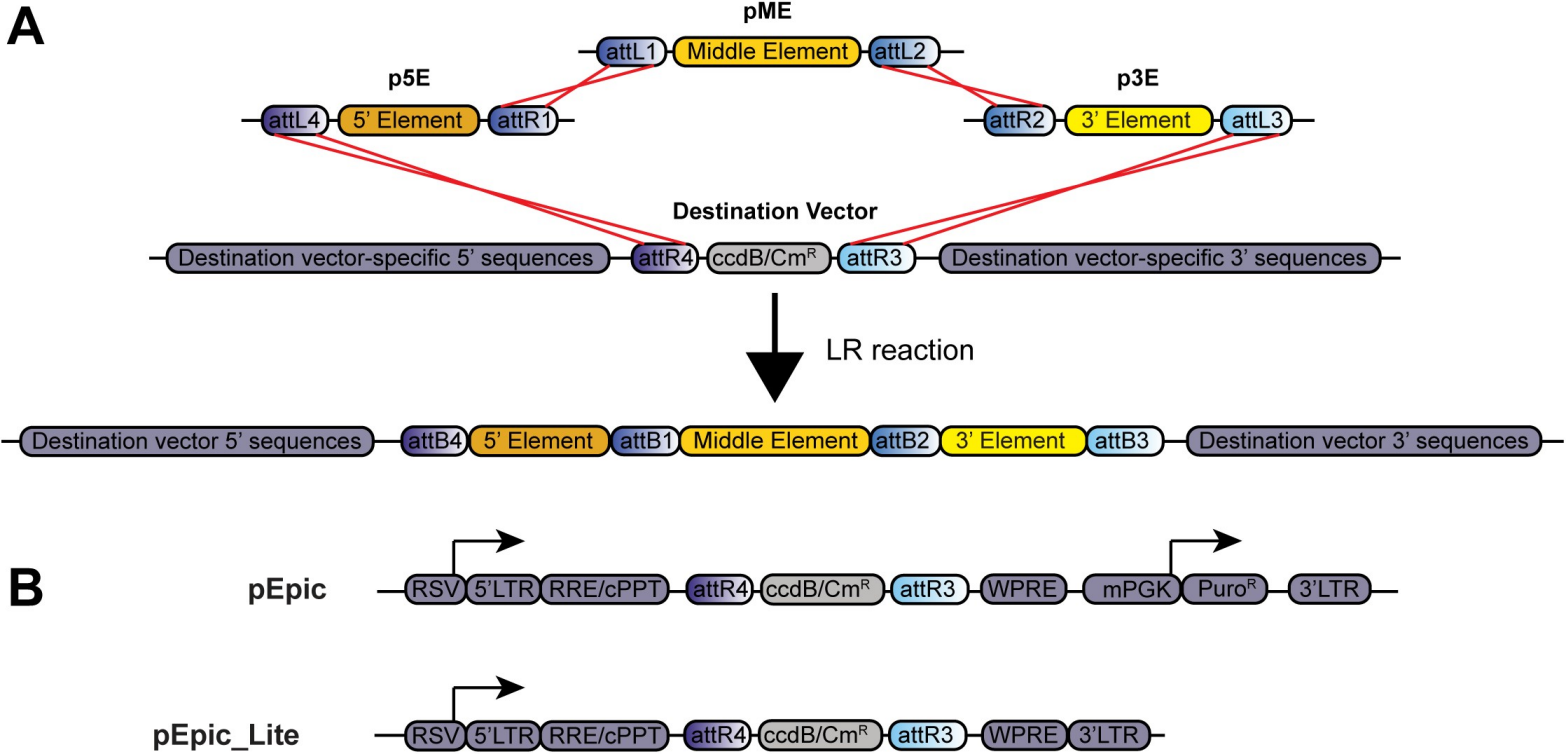
Overview of three-fragment MultiSite Gateway cloning and novel lentiviral destination vectors. (A) Schematic of an LR recombination reaction and the resulting vector. Site-specific recombination events (red lines) between attR and attL sites from a 5’, middle, and 3’ entry vector with a destination vector replaces the ccdB/Cm^R^ selection cassette of the destination vector with the mobile DNA elements from the entry vectors, leaving destination vector-specific 5’ and 3’ sequences intact. (B) Schematic of lentiviral destination vectors pEpic and pEpic_Lite. attR3 and 4 sites flanking the ccdB/Cm^R^ selection cassette are positioned in an anti-sense orientation to viral RNA expression driven by a Rous sarcoma virus (RSV) promoter. pEpic_Lite lacks puromycin resistance (Puro^R^). LTR = long terminal repeat; RRE = Rev response element; cPPT = central polypurine tract; ccdB = E. coli ccdB toxin; Cm^R^ = chloramphenicol resistance; mPGK = mouse phosphoglycerate kinase promoter; WPRE = woodchuck hepatitis virus posttranslational regulatory element.

The correct plasmids and their sequences have been deposited with Addgene (www.addgene.org), with the following catalog numbers: pEpic #84372; pEpic_Lite #84373.
